# Control of the Singlet–Triplet Energy Gap in a Thermally Activated Delayed Fluorescence Emitter by Using a Polar Host Matrix

**DOI:** 10.1186/s11671-017-2012-1

**Published:** 2017-04-11

**Authors:** Shota Haseyama, Akitsugu Niwa, Takashi Kobayashi, Takashi Nagase, Kenichi Goushi, Chihaya Adachi, Hiroyoshi Naito

**Affiliations:** 1 Department of Physics and Electronics, Osaka Prefecture University, 1-1 Gakuencho, Naka, Sakai, 599-8531 Japan; 2 Research Institute of Molecular Electronic Devices (RIMED), Osaka Prefecture University, 1-1 Gakuencho, Naka, Sakai, 599-8531 Japan; 3 Center for Organic Photonics and Electronics Research (OPERA), Kyushu University, 744 Motooka, Nishi, Fukuoka, 819-0395 Japan; 4 Adachi Molecular Exciton Engineering Project, ERATO, Japan Science and Technology Agency (JST), 744 Motooka, Nishi, Fukuoka, 819-0395 Japan

**Keywords:** Thermally activated delayed fluorescence (TADF), Photoluminescence spectroscopy, Electronic permittivity, Polar host matrix

## Abstract

The photoluminescence properties of a thermally activated delayed fluorescence emitter, 1,2-bis(carbazol-9-yl)-4,5-dicyanobenzene (2CzPN), doped in a host matrix consisting of 1,3-bis(9-carbazolyl)benzene and a polar inert molecule, camphoric anhydride (CA), in various concentrations have been investigated. It is found that the addition of CA stabilizes only the lowest singlet excited state (S_1_) of 2CzPN without changing the energy level of the lowest triplet excited state (T_1_), leading to a reduction in the energy gap between S_1_ and T_1_. The maximum reduction of energy gap achieved in this work has been determined to be around 65 meV from the shift of the fluorescence spectrum and the temperature dependence of the photoluminescence decay rate.

## Background

In recent years, materials that exhibit thermally activated delayed fluorescence (TADF) have received considerable attention because of their strong potential to realize highly efficient and low-cost organic light-emitting diodes (OLEDs) [1, 2]. One of the important factors that determine the efficiency of TADF is the energy gap between the lowest singlet (S_1_) and triplet (T_1_) excited states (Δ*E*_ST_), which should be small enough that the generated triplet excitons overcome it by thermal energy at room temperature to be converted into singlet excitons. It is known that Δ*E*_ST_ can be reduced by spatially separating the highest occupied molecular orbital (HOMO) and lowest unoccupied molecular orbital (LUMO) [3, 4]. In fact, such charge-transfer (CT) molecules have been reported to exhibit efficient TADF [5–10]. When a CT excited state is created by, for example, photoexcitation, a change in the permanent dipole moment is induced. In a polar solvent, the reorientation of the polar solvent molecules stabilizes the CT excited state, and consequently, the photoluminescence (PL) spectrum shifts with respect to that in a non-polar solvent [11]. Some CT molecules also have a locally excited (LE) state, which is not stabilized even in a polar solvent, and thus, the energy gap between the CT and LE states and even their order may change depending on the polarity of the solvent used [11]. Such solvent polarity effects have been investigated in some TADF emitters [8, 12, 13]. Ishimatsu et al. have determined the Δ*E*_ST_ values of a TADF emitter in several solvents and found that the Δ*E*_ST_ value reduces as the solvent polarity increases [13]. This result suggests that the originally small Δ*E*_ST_ value could be further reduced by doping the TADF emitter in a polar host matrix. Although TADF emitters are used in solid state in OLEDs, Madian et al. have confirmed that a spectral shift of the solid-state PL similar to that observed in a polar solvent can be induced by using a two-component host consisting of an inert polymer and a polar inert molecule, camphoric anhydride (CA) [14]. Therefore, the addition of a polar inert molecule could be another strategy to achieve a small Δ*E*_ST_ value. In this work, to examine the effectiveness of this strategy, we investigated the PL properties of a TADF emitter, 1,2-bis(carbazol-9-yl)-4,5-dicyanobenzene (2CzPN), doped in host matrixes with different electronic permittivities, which are controlled by adding CA in various concentrations.

## Methods

**Fig. 1:**
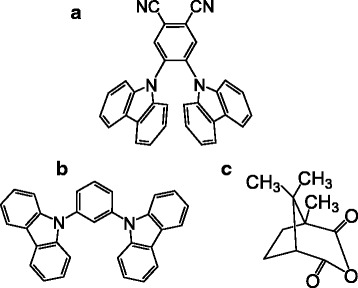
Chemical structures of **a** 2CzPN, **b** m-CP, and **c** CA

We used 1,3-bis(9-carbazolyl)benzene (m-CP) as the main host material. Thin films of m-CP with 2CzPN-doping concentration of 5 wt% were spin coated from dichloromethane solutions onto sapphire substrates. The prepared thin films were around 100 nm thick. To control the permittivity of the thin films, we added CA to m-CP in various concentrations while keeping the 2CzPN-doping concentration against the total host matrix constant (5 wt%). Hereafter, we only show the weight ratio of m-CP:CA, which was changed from 100:0 (0 wt%) to 50:50 (50 wt%). The chemical structures of 2CzPN, m-CP, and CA are shown in Fig. 1. Steady-state PL spectra were recorded using a He–Cd laser (λ = 325 nm) and an optical multichannel analyzer with a calibrated CCD. For time-resolved PL measurements, we used the third harmonic wavelength of a Nd:YAG laser (*λ* = 355 nm) as a pulsed excitation source and recorded PL decay curves with a photomultiplier tube and a photon counting system (a multichannel scaler). A phosphorescence spectrum was recorded at 6.5 K by integrating PL in a time range from 30 to 1000 ms after pulsed excitation. Note that m-CP and CA have large band gaps and are thus transparent at those excitation wavelengths. Electronic permittivity measurements were performed using an impedance analyzer on devices with Al (40 nm)/2CzPN:m-CP:CA (100 nm)/Al (40 nm) structure. The active device area, which was defined by a metal mask for Al deposition, was 1.44 mm^2^.

## Results and Discussion

**Fig. 2:**
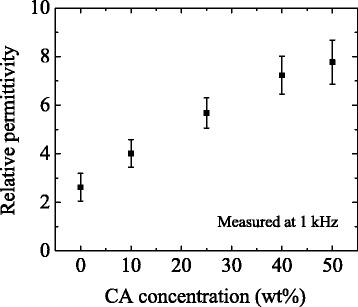
Plot of the relative permittivity of the prepared devices versus CA concentration. The permittivity was almost constant in the 10–10^5^ Hz frequency range, and the data taken at 1 kHz are plotted here

**Fig. 3:**
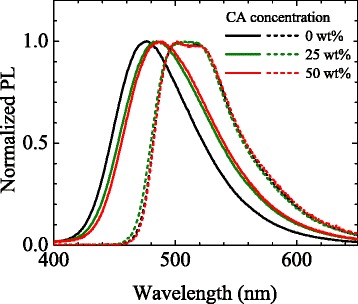
Fluorescence (*solid lines*) and phosphorescence (*dashed lines*) spectra of 2CzPN-doped m-CP:CA thin films. *Black*, *green*, and *red lines* represent the data for CA concentrations of 0, 25, and 50 wt%

The measured permittivities of the prepared devices are shown in Fig. 2. The permittivity increases linearly with increasing CA concentration up to 40 wt%. Although the permittivity seems to slightly saturate above 40 wt%, the results confirm that the permittivity can be controlled in a wider range than that reported before [14]. As shown in Fig. 3, as the CA concentration increases, the fluorescence spectrum of 2CzPN is gradually redshifted. Since the fluorescence of 2CzPN originates from S_1_, the redshift indicates stabilization of S_1_ in higher CA concentrations. On the other hand, the phosphorescence spectrum is virtually independent of permittivity. This suggests that T_1_ is not a CT state but an LE state. The observation that the phosphorescence spectrum is narrower than the fluorescence spectrum also supports this assignment. Because of the different characters of S_1_ and T_1_, the addition of CA results in the reduction of Δ*E*_ST_ in 2CzPN.

**Fig. 4:**
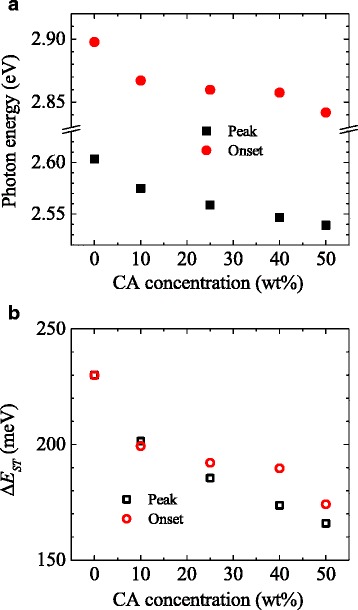
**a** Spectral position of fluorescence and (**b**) estimated Δ*E*_ST_ as a function of CA concentration. In panel **a**, *solid squares* and *circles* represent the peak photon energy and the onset of fluorescence, respectively. In panel **b**, *open squares* and *circles* indicate Δ*E*_ST_ values estimated from the peak photon energy and the onset, respectively, under the assumption that Δ*E*_ST_ is 230 meV at 0 wt% CA concentration

In Fig. 4a, we plot the peak photon energies of the prepared thin films as a function of the CA concentration. This figure more clearly shows that the fluorescence of the prepared thin films is gradually redshifted as the CA concentration increases. In Fig. 4b, we show the Δ*E*_ST_ values determined from the difference between the peak photon energies of the fluorescence and phosphorescence under the assumption that Δ*E*_ST_ = 230 meV (discussed in detail below) for 5 wt% 2CzPN-doped m-CP thin films (i.e., 0 wt% CA concentration). The figure shows that the reduction of Δ*E*_ST_ reaches 65 meV at a CA concentration of 50 wt%. Note that since the observed fluorescence and phosphorescence do not have an identical spectral shape, the Δ*E*_ST_ values determined from the peak photon energy might have a large error. Hence, we also have determined Δ*E*_ST_ values from the difference in the onsets between fluorescence and phosphorescence. These data are also plotted in Fig. 4. The maximum reduction of Δ*E*_ST_ determined from the onsets is slightly smaller but is still 56 meV.

**Fig. 5:**
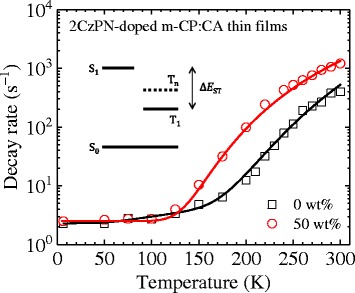
Temperature dependence of the decay rate of 2CzPN-doped m-CP:CA thin films. *Solid lines* are fitted results based on a four-level model shown in the *inset*

There are several ways to determine Δ*E*_ST_ in TADF emitters. The simplest way is, as demonstrated above, to determine it from the difference in the peak photon energy or the difference between fluorescence and phosphorescence onsets [15]. Recently, we had proposed another way to estimate Δ*E*_ST_ by analyzing the temperature dependence of the decay rate of the most slowly decaying PL component with a four-level model including an additional triplet excited state (T_n_) lying between S_1_ and T_1_ [16]. In the model, we have assumed that the thermal activation processes from T_n_ and T_1_ can be described by a Boltzmann distribution. To apply this way to 2CzPN, we measured the temperature dependence of the decay rate of 2CzPN-doped m-CP:CA thin films with CA concentrations of 0 and 50 wt% (see Fig. 5). In Fig. 5, we also show the best fits to the four-level model. From the fits, the energy gaps between S_1_ and T_1_ and between T_n_ and T_1_ are determined, as shown in Table 1. If the former gap is regarded as Δ*E*_ST_, its maximum reduction due to the addition of CA is again determined to be around 65 meV. In contrast to T_1_, the energy level of T_n_ is slightly lowered. This fact suggests that T_n_ has more CT character than T_1_.

**Table 1:** Energy gaps determined with a four-level model including a higher triplet excited state (T_n_)

Energy gap	0 wt%	50 wt%
S_1_–T_1_	230 ± 10 meV	165 ± 10 meV
T_n_–T_1_	150 ± 5 meV	120 ± 5 meV

**Fig. 6:**
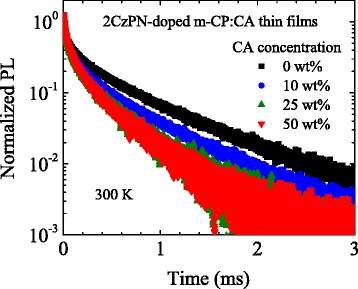
PL decay curves measured at 300 K for 2CzPN-doped m-CP:CA thin films with several CA concentrations

In Fig. 6, we show the PL decay curves of some of the prepared thin films at room temperature. The slowest decay rates at 300 K in Fig. 5 are obtained by fitting a sum of several exponential functions to those decay curves. It is found that the PL decay at 300 K is enhanced by addition of CA. At room temperature, the slowest decay rate is mainly determined by the thermal activation process from T_1_ to S_1_. Therefore, the acceleration of this thermal activation process can be regarded as another evidence showing the decrease in Δ*E*_ST_. Note that the acceleration of the decay rate due to the addition of CA is seen not only at room temperature but also at all temperatures above 150 K (see Fig. 5). TADF emitters have a relatively slow decay rate due to the delayed fluorescence. Therefore, under a high current injection condition, the density of the excited states tends to significantly increase, and thus, an unfavorable exciton–exciton annihilation process such as singlet–triplet annihilation and triplet–triplet annihilation [17, 18] may reduce the external quantum efficiency (EQE). This phenomenon is known as the efficiency roll-off. The addition of CA or another polar host material may also be a solution to suppress the reduction of EQE due to the exciton–exciton annihilation process.

## Conclusions

We have demonstrated that the Δ*E*_ST_ of 2CzPN can be reduced by increasing the permittivity of the host matrix by CA doping. The maximum reduction in Δ*E*_ST_ at a CA concentration of 50 wt% is determined to be 56–65 meV from the analysis of the temperature dependence of PL decay rate as well as the spectral difference in the fluorescence and phosphorescence spectra. The reduction is achieved because only S_1_ is stabilized in the host with a larger permittivity. This method is, therefore, effective only for TADF emitters having an LE state as T_1_. Reduction of Δ*E*_ST_ accelerated the thermal activation process from T_1_ to S_1_, thereby reducing the triplet exciton density; the latter is expected to contribute to the suppression of the efficiency roll-off.

## Abbreviations

2CzPN: 1,2-bis(carbazol-9-yl)-4,5-dicyanobenzene
CA: Camphoric anhydride
CT: Charge-transfer
EQE: External quantum efficiency
HOMO: Highest occupied molecular orbital
LE: Locally excited
LUMO: Lowest unoccupied molecular orbital
m-CP: 1,3-bis(9-carbazolyl)benzene
OLEDs: Organic light-emitting diodes
PL: Photoluminescence
TADF: Thermally activated delayed fluorescence
